# Partner notification and STI patient experiences in Ekurhuleni East, Gauteng, South Africa

**DOI:** 10.4102/phcfm.v17i1.4904

**Published:** 2025-06-09

**Authors:** Noluthando Mpobole, Raikane J. Seretlo, Mathildah M. Mokgatle

**Affiliations:** 1 Department of Public Health, School of Health Care Sciences, Sefako Makgatho Health Sciences University, Pretoria, South Africa

**Keywords:** sexually transmitted infections, partner notification practices, sexual partners, Ekurhuleni East sub-district, experiences

## Abstract

**Background:**

Sexually transmitted infections (STIs) are a widespread public health concern, disproportionately impacting young adults. This group is at higher risk due to factors like limited knowledge, misconceptions about sexual health, inconsistent condom use, multiple sexual partners, and barriers to youth-friendly sexual and reproductive health services. Partner notification (PN) strategies are critical in STI control. Effective notification and communication with sexual partners can help break the infection chain and encourage early diagnosis and treatment. However, the dynamics of PN in young individuals remain unclear.

**Aim:**

To explore PN practices and experiences of STI-diagnosed patients with their sexual partners in the Ekurhuleni East sub-district, Gauteng, South Africa.

**Setting:**

The study was conducted in five healthcare facilities in Ekurhuleni East.

**Methods:**

Thirty participants were selected purposively, using an exploratory design. A semi-structured interview guide and in-depth face-to-face interviews were used. Data were analysed using thematic content analysis, following Braun and Clarke’s six-phase approach: familiarisation, coding, theme identification, theme review, theme definition, and report production.

**Results:**

The study identifies key areas for improving STI prevention and PN. Low understanding and cultural attitudes around STIs highlight the need for culturally relevant health education. PN presents emotional and relational issues, including intimate partner abuse and casual relationships, indicating the need for supportive and adaptable notification methods.

**Conclusion:**

The study highlights the importance of addressing knowledge gaps and cultural beliefs about STIs and promoting open communication in relationships.

**Contribution:**

This study contributes insights to healthcare policymakers and practitioners to improve STI management and safer sexual practices among young people.

## Introduction

Partner notification (PN) refers to the practice of alerting sex partners of patients with sexually transmitted infections (STIs) that they may have been exposed to an infection, and that they should seek medical attention.^1^ Partner notification facilitates early diagnosis of sexual partners and encourages behaviour modification in both clients and their partners. It also helps to lower the overall disease burden within communities. In addition, PN provides index patients with an opportunity to educate their partners about risk-reduction strategies for preventing STIs.^2^ Young people generally struggle with STI PN and treatment.^3^ It is further stated that novel techniques are required to overcome obstacles in the STI care cascade.^3^

In addition, PN is as a strategy can assist in lowering STI rates.^2^ It is recommended that this action should be voluntary and should take place within appropriate social and legal contexts.^4^ According to Ward and Bell,^5^ when notifications are handled correctly, they can help in identifying asymptomatic sexual infections, lower the risk of infection transfer, avoid sequelae and provide a forum for talking about safe sex.

Although patients diagnosed with STIs have several options for notifying their partners, the effectiveness of these methods depends largely on individual preferences.^1^ If people are uncomfortable with the method provided, they are less likely to inform their partners.^6^

Studies conducted among global and African university students indicate a general lack of awareness and belief in STIs or human immunodeficiency virus or acquired immunodeficiency syndrome (HIV/AIDS). Reuter et al.^7^ argue that when individuals are not taking action to protect themselves against these diseases, they potentially increase their risk of contracting these diseases. Moreover, a study conducted by Alam et al.^8^ on PN of STI found that reported barriers to PN included sociocultural factors such as stigma, fear of abuse and infrastructural factors related to the limited number of STI clinics, trained providers and reliable diagnostic methods. Although this is the case, client-oriented counselling effectively improves partner referral outcome.

In 2016, the World Health Assembly approved a worldwide health sector strategy on STIs for 2016–2021, and it included the ambitious 2020 and 2030 goals that are in line with more general sustainable development goals as well as targets to eliminate disease outbreaks as public health issues.^9^ The management of STIs depends heavily on PN. As part of syndromic management or following the findings of an STI test, the process includes identifying exposed sex partners, informing them that they have been exposed to a curable STI and providing counselling and treatment.^10^ Its objectives are to stop the index patient from contracting a treatable STI from a partner who has not received treatment, lessen the burden of curable STIs in the community and prevent adverse health effects in both the index patient and his or her sex partners.^1^

According to a global study, digital PNs are now an option, although it is unclear whether people accept the communication.^11^ In addition, specific communities may find some approaches more acceptable than others because of their attitudes and beliefs on sexual behaviour and STIs.^12^ Patient delivered partner therapy (PDPT) is the practice of giving medication for treatment to the spouses of the patients diagnosed with bacterial STI without requiring the partner to participate in diagnostic tests and counselling.^13^

Among the PN intervention strategies, counselling of index STI patients and patient-delivered partner medication were somewhat effective in Africa.^10^ According to Ferreira et al.,^1^ there are mechanisms in place for PN, including patient referral, expedited partner therapy and referral of provider contact. Furthermore, a study conducted in Republic of South Africa indicated that a critical feature of the syndromic management of STIs is PN, which uses notification slips provided during consultations with healthcare professionals.^14^

## Purpose

The growth in STI cases among young people is concerning for public health, and one method for preventing and reducing further transmissions is to tell sexual partners and complete treatment after an STI diagnosis.^15^ The prompt identification and appropriate treatment of all sexual partners of patients with STIs are required for the effective management of STIs.^16^ Hence, the aim of this study was to explore PN practices and experiences of STI-diagnosed patients with their sexual partners in the Ekurhuleni East sub-district, Gauteng, South Africa. The results from the study were anticipated to shed light on the barriers and facilitators to PN practices and providing insights that can be used to enhance public health initiatives and educational programmes to empower young adults to make informed sexual health decisions.

## Research methods and design

The study employed an exploratory qualitative descriptive design to obtain in-depth information about the experiences of young adults regarding PN. The researcher chose this design to explore PN practices and the experiences of patients diagnosed with STIs in relation to their sexual partners.

### Research setting and context

The research was carried out at Ekurhuleni Health District. It is one of five districts in the Gauteng province, South Africa, East of Johannesburg and reaching the Mpumalanga border. Ekurhuleni is primarily an urban and sub-urban area within Gauteng province, South Africa. It includes a mix of formal residential areas, large, urbanised townships and a significant number of informal settlements. The district is divided into three geographical sub-districts: North, South and East. The study was conducted in five healthcare facilities, four community health centres (CHCs) that operate 24 h and one clinic that operates from 07:30 to 16:30 weekdays at Ekurhuleni East sub-district.

### Study population and sampling

The study was conducted among 30 participants from the selected five healthcare facilities, in the Ekurhuleni East sub-district. Purposive sampling was used for this study. The researcher chose this method to gain detailed information and knowledge about STI PN practices and experiences of young people diagnosed with STIs. Data saturation was reached at 24 interviews when STI clients gave similar responses and no longer received new information; however, the researcher continued to confirm data saturation by conducting an additional six interviews.^17^ As a result, the study had 30 participants in total.

### Inclusion criteria

The inclusion criteria consisted of both male and female patients between the ages of 18 and 39 years who had been diagnosed with STIs and must be or have received healthcare services from the selected study settings. This age group was selected because young adults are disproportionately affected by STIs because of factors such as increased sexual activity, multiple partners, inconsistent condom use and limited access to comprehensive sexual health education and services. Focusing on this demographic allows for a deeper understanding of the behaviours, experiences and challenges they face regarding STI prevention and partner notification.

### Exclusion criteria

Clients who were not willing to participate in or who did not give consent were excluded. All participants who had not received healthcare services from the selected study settings were also excluded.

### Data collection

Data were collected from July 2024 to August 2024. The first three interviews were considered for a pilot study, the purpose of which was to test the questions in the interview guide to see whether they would help to answer the research questions and get the required information, thus testing the procedure, the tool and the process. A semi-structured guide was used during the in-depth face-to-face interviews to collect data. The interviews were guided by an interview guide, which included demographic details, main questions and probing questions based on participants’ responses to ensure in-depth data were obtained (see Table 1 for interview questions). Moreover, field notes were recorded during the interviews to capture participants’ non-verbal expressions.

**TABLE 1:** Interview questions.

Questions	Probes
What is your understanding about STIs?	In your own words, please explain to me what would you say STIs are? Could you please explain to me how does one get STIs? Please tell me the symptoms of STIs?
Please tell me is there anything you would like to share regarding the time you were diagnosed with STI?	Would you like to share with me how did you feel when you were diagnosed with STIs? Were you aware of the type of STI that you had?
Would you like to share with me what it was like when you informed your partner about the STI, at the time you were diagnosed?	Could you please tell me how did your partner find out? Are you not informing them? How did you inform your partner when you found out about STI? Could you explain the reason why you did not inform or disclose to them about STI?
Would you like to share with me how did you inform your partner after finding out that you have STI?	Could you please share with me how did your partner take it? Would you like to share more about how did your partner reacted when you told them about STI? Please share with me, what were the other reactions that your partner made? Could you please share with me, how did that make you feel? Did you give him or her anything from the clinic? What were the messages from the nurse for you to give to your partner? Could you please tell me what your partner’s response was?

STIs, sexually transmitted infections.

Both verbal and written informed consent were obtained from participants who were willing to participate before the interview process. A voice recorder was used during individual interviews, and each interview lasted for about 30-45 min. The recorded interviews were transcribed verbatim, and the Zulu audios were transcribed into English by the researcher.

### Data analysis

The thematic content analysis followed Braun and Clarke:^18,19^ (1) familiarising yourself with the data, (2) generating initial codes, (3) searching for themes, (4) reviewing themes, (5) defining and naming themes, and (6) producing the report six-phase theme analysis approach. The socio-demographic data were summarised using an Excel spreadsheet. The researcher first became familiar with the data by listening to all of the audio recordings while reading the transcripts to ensure the completeness of the data. The researcher generated initial codes independently, developed a manual code book for the first three interviews from the main study and made notes on the reflections from the data. Subsequently, the researcher searched for the themes by identifying similarities in data, reviewed the themes by developing, interrogating and evaluating themes for similarities and differences in meaning and developed themes and sub-themes. The transcripts were imported to NVivo 14. All transcripts were coded as they emerged and were recorded as initial coding on NVivo 14. Once the coding was completed, the researcher began organising the codes into broader themes. Digital recordings of the interviews were professionally transcribed verbatim. Data were analysed in collaboration with the co-supervisor; the final phase of data analysis involved writing up the results from the themes and supporting them with participant quotes to ensure trustworthiness of the findings.

### Trustworthiness

To ensure trustworthiness, the researcher followed Lincoln and Guba^20^ ‘quality criteria framework’ and applied the following principles: credibility, dependability, confirmability and transferability. To ensure credibility, the researcher established confidence in the study phenomena through peer debriefing from the co-supervisor and spent time engaging with the participants throughout the interviews to foster trust and to guarantee that in-depth, valuable data were obtained. Dependability was ensured by keeping an audit trail from detailed documentation of the procedures and decisions made throughout the study. The researcher established confirmability by utilising measures to minimise personal bias and ensuring that the findings represented the participants’ responses. The findings were linked directly to the data by providing direct participant quotes to support the themes. To ensure transferability, the researcher ensured that data analysis was done precisely, consistently and exhaustively by recording and systematising everything.

### Ethical considerations

Ethical approval to conduct this study was obtained from the Sefako Makgatho Health Sciences University Research Ethics Committee (SMUREC) (No. SMUREC/H/474/2023:PG) and Ekurhuleni Health District Research Committee (EHDRC). Permission to access Ekurhuleni East sub-district healthcare facilities was accorded by the East Primary Health Care (PHC) sub-district manager.

After receiving the final permission from the sub-district PHC manager, appointments with different and relevant facilities’ managers were made telephonically. For some healthcare facilities, the researcher personally went to make appointments for interviews.

As the research consisted of human subjects as participants because of the nature of the research, the following ethical considerations were employed throughout the research process. All participants were informed about their expectations, purpose and objectives of the study and were given a consent form. They were explained about the importance of their right to withdraw at any time during the research process without consequences. Participants’ names were not included in the recording and writing. Codes were used instead of participant and facility names to safeguard participants’ anonymity. Field notes, recordings and informed consent forms were kept in a locked cupboard, and any information transcribed in a computer was password protected to ensure the privacy and confidentiality of the participants. Privacy was ensured by conducting individual interviews in a private room. Additional measures were taken to help participants feel comfortable, such as reassuring them of confidentiality, using empathetic and non-judgemental language, allowing them to choose the interview time and creating a safe, respectful environment for open communication. Because the audio recorder was used, it was wiped with sanitiser or an alcohol-based cloth after every participant interview to prevent cross-infection.

## Results

Data were collected from a sample of 30 participants (20 females and 10 males). Participants were between the age of 18 and 39 years: 10 between the age of 30–34 years, 9 between the age of 35–39 years, 6 between the age of 25–29 years and 5 between the age of 18–24 years. Table 2 illustrates all the demographic information of the 30 participants.

**TABLE 2:** Demographic information of the study participants (*N* = 30).

Participant number	Age (years)	Gender	Relationship status
Participant 1	32	Female	In a relationship
Participant 2	32	Female	In a relationship
Participant 3	35	Female	In a relationship
Participant 4	35	Female	Married
Participant 5	31	Female	Single
Participant 6	33	Female	In a relationship
Participant 7	36	Male	In a relationship
Participant 8	35	Female	In a relationship
Participant 9	37	Female	Married
Participant 10	32	Female	In a relationship
Participant 11	33	Female	In a relationship
Participant 12	29	Female	In a relationship
Participant 13	22	Female	In a relationship
Participant 14	32	Female	In a relationship
Participant 15	39	Male	Single
Participant 16	20	Male	Single
Participant 17	22	Male	In a relationship
Participant 18	32	Male	Single
Participant 19	19	Female	In a relationship
Participant 20	22	Female	In a relationship
Participant 21	25	Female	In a relationship
Participant 22	36	Male	In a relationship
Participant 23	25	Male	In a relationship
Participant 24	39	Male	In a relationship
Participant 25	31	Female	In a relationship
Participant 26	26	Male	In a relationship
Participant 27	29	Male	In a relationship
Participant 28	26	Female	In a relationship
Participant 29	35	Female	In a relationship
Participant 30	31	Female	In a relationship

Two main themes emerged from the study with related five sub-themes. The themes and sub-themes that reflect PN practices and experiences among patients diagnosed with STIs are summarised in Table 3.

**TABLE 3:** Summary of themes and sub-themes.

Main themes	Sub-themes
1. Awareness and knowledge regarding STIs	1.1 Understanding of STI transmission, prevention and symptoms
2. Notification practices when informing their partners	2.1 Various experiences when notifying their partners 2.2 Relationship dynamics (intimate partner violence, infidelity, casual sex or one-night stand relationships) 2.3 Emotional responses of participants during PN (anger, fear, shame, self-blame and disappointment in partner) 2.4 Methods of notifying partners

STIs, sexually transmitted infections; PN, partner notification.

### Theme 1: Awareness and knowledge regarding sexually transmitted infections

Theme 1 revealed the study participants’ understanding of the mode of STI transmission, how STIs can be prevented and how to determine whether one is infected. The theme included participants’ responses on how different beliefs contribute to the awareness of STI treatment methods.

#### Sub-theme 1: Understanding of sexually transmitted infection transmission, prevention and symptoms

The participants were asked about their understanding and knowledge regarding STI. Most had a sense of how they could be infected and indicated that one could be infected if having sexual intercourse with women who are menstruating, having sex without a condom and having sex with someone who has multiple sexual partners. The following quotes highlight this further:

‘My understanding about STI is that you get it when you sleep with an infected person or when you are on periods.’ (Participant 20, 22-year-old female)

‘It is different, … you can get it sleeping with a guy who sleeps around, from a guy who is not circumcised, not using a condom. Someone who sleeps around will get STI and bring it to you and infect you.’ (Participant 19, 19-year-old female)

‘The first one is burning urine. When you get to the toilet, there is no urine coming out. It’s just painful, then all of a sudden, your underwear is wet, you don’t know how. The other one is itching, and you get small pimples.’ (Participant 16, 20-year-old male)

‘They are different, and sometimes there can be genital warts and rash, or that it is itchy on the private part.’ (Participant 8, 35-year-old female)

‘Sometimes you will have this liquid that comes from your vagina. The discharge! Yes.’ (Participant 8, 35-year-old female)

‘I can say … I do not use a condom, then I’ll get an STI like that … my vagina was itching, I also had a discharge that was not clean, and it had an odour.’ (Participant 9, 37-year-old female)

‘I used a condom when my partner comes back; he loves sex, so I give it to him, but we use a condom.’ (Participant 29, 35-year-old female)

‘I understand that, as women, we need to protect ourselves because when someone has diseases, they do not want you to know that they are sick; they infect you. Now, we need to get used to using condoms so that we can be safe.’ (Participant 5, 31-year-old female)

‘I must use a condom when having sex.’ (Participant 20, 22-year-old female)

### Theme 2: Notification practices when informing their partners

This theme emerged when participants indicated the notification practice they used to inform their partners, as well as their experiences while notifying their partners about an STI diagnosis. It revealed different relationship dynamics and further revealed various emotional responses. In addition, it explored other methods of informing partners. Participants shared their challenges and partners’ reactions, from support and cooperation to blame and denial.

#### Sub-theme 1: Various experiences when notifying their partners

Different sensitive experiences were expressed by the participants when they had to inform their partners. Further, participants’ experiences ranged from fear of blame to conflict. Some found it challenging to initiate the conversation, while others received support or were met with resistance. Several participants described feeling apprehensive about informing their partners, anticipating blame, accusations of infidelity or denial of the diagnosis. This is illustrated in the following quotations:

‘I was very frustrated, to be honest with you. I even stopped monitoring this thing because it was just bringing fights into my house; there was no peace.’ (Participant 10, 32-year-old female)

‘It was a difficult day for us, and the fact that it says sexually transmitted, automatically he said I was cheating.’ (Participant 2, 32-year-old female)

‘I was afraid he would say I am sleeping around and might blame me. Men tend to want to be defensive. So, I thought, even though it happened when I told him, but at the end, he agreed to consult.’ (Participant 9, 37-year-old female)

In some cases, participants were able to discuss the diagnosis openly with their partners, who responded positively and agreed to seek treatment:

‘I told him that it is urgent. He must urgently go to the clinic so that he can be treated. Luckily, he listened to me and went to the clinic.’ (Participant 3, 35-year-old female)

However, during the notification to their partners, several participants experienced resistance or complete denial from their partners, who refused to seek treatment because they were not the ones who were diagnosed and, in some instances, participants reported that they were blamed by them for the infection:

‘According to him, I am the one with this. I am the one sick, not him, and he won’t go to the clinic.’ (Participant 13, 22-year-old female)

‘So, he said I am dating guys from the streets. He was very, very angry, refusing to go to the clinic.’ (Participant 2, 32-year-old female)

‘He said he does not have any symptoms. I’m the one who’s sick, then it means I’m the one who came with the STI.’ (Participant 28, 26-year-old female)

#### Sub-theme 2: Relationship dynamics

This theme emerged when participants were asked to reflect upon the time they informed their partners about their STI diagnosis. Participants indicated the impact of relationship dynamics on their experiences with PN and their responses. Participants highlighted how intimate partner violence (IPV), infidelity and casual sexual relationships influenced their ability to communicate about STIs, often complicating or hindering effective PN.

**Intimate partner violence:** For some participants, disclosing an STI diagnosis resulted in verbal or physical abuse, making it challenging and even dangerous to communicate openly with their partners. Fear of blame, accusations and violence added emotional distress, creating barriers to honest discussions about STIs. Participants’ responses are quoted as follows:

‘Uhm! My God! Ei! When I told him the first thing, he told me about the way I am sleeping around, how I got it, and now I am going to infect him. He beat me all night; we did not sleep.’ (Participant 13, 22-year-old female)

‘He was very angry because he turned everything around and said I do not trust him, so that is why I want him to test, and he won’t go because he is fine, and his wife is fine. It meant the problem was with me.’ (Participant 30, 31-year-old female)

‘Hey! I blocked him (telephone contact) because he was insulting me. I said that you have it, that is why you’re insulting.’ (Participant 20, 22-year-old female)

**Infidelity:** Infidelity emerged as a significant issue affecting PN. Some participants knew or suspected that their partners were unfaithful, which led to mistrust, accusations and resistance to treatment. Participants stated:

‘He treated it his way because I remember we spent about two weeks not talking to each other. We were fighting, we had trust issues.’ (Participant 12, 29-year-old female)

‘The person I am in a relationship with, I discovered he had someone else. He is married and wants polygamy. I saw that it is important to have one partner because I was dating only him, but he had his partner.’ (Participant 30, 31-year-old female)

‘Not a pleasant one, we had a little fight because we did not know who brought the STI into the relationship. Yeah, … I was being accused of everything.’ (Participant 26, 26-year-old male)

**Casual sex or one-night-stand relationships:** Participants who were in casual relationships or had one-night stands presented different challenges in terms of STI notification. In these instances, participants often did not feel obligated to inform their partners or were unwilling to discuss the matter because of the temporary nature of these relationships. Participants highlighted in this context:

‘It was only something for that time; I won’t meet with her again, and it was a mistake – not that I did it on purpose. A condom broke.’ (Participant 18, 32-year-old male)

‘Well, it was not my partner. I never told her. I did not need to tell her because we were no longer together. If her partner gets it, he will tell her, then they will go together.’ (Participant 16, 20-year-old male)

‘I used to go to pubs and have drinks with friends. Sometimes, we find out that you hooked another person for that night, and it’s difficult to use a condom during sex while you are drunk. So that’s why I may say I was unaware of where I acquired the STI.’ (Participant 22, 36-year-old male)

#### Sub-theme 3: Emotional responses of participants during partner notification

This theme merged when participants were asked how they felt when they were diagnosed with STI and how their partner’s responses or reactions to notification made them feel. Participants indicated a range of emotions. Emotions such as anger, fear, shame, self-blame and disappointment:

‘I was angry with my husband.’ (Participant 4, 35-year-old female)

‘Ei! It changed my mind about our relationship; it made me feel like I am not safe. One day I will wake up having HIV, because after that I called him, he came I told him that I bought test kits for HIV, so we need to test. He told me that No my first wife is pregnant, and she tested negative it means that I am also negative.’ (Participant 30, 31-year-old female)

‘I was ashamed because I am married.’ (Participant 9, 37-year-old female)

‘I was feeling like a bad partner, … I was feeling like the person I’m with doesn’t even deserve to be with someone like me.’ (Participant 23, 25-year-old male)

‘What hurts me is that I have a child with this person. Why is he doing something like this?’ (Participant 5, 31-year-old female)

#### Sub-theme 4: Methods of notifying partners

Participants adopted different strategies to inform their partners about their STI diagnosis, reflecting clinic guidance and personal comfort levels. The approaches included notification slips provided at the healthcare facility, face-to-face conversations and telephone notifications. Several participants indicated that they received formal notification slips from clinics to give to their partners, which was an official request for their partners to seek testing and treatment. Their responses included statements such as:

‘There was a letter she (the sister) wrote for me to give my partner. I explained to him he didn’t even want to read what was written.’ (Participant 2, 32-year-old female)

‘At the clinic, they gave me a PN slip. I took it home, and then when my partner came, I informed him.’ (Participant 28, 26-year-old female)

In addition, some participants indicated direct in-person discussions as another method, as some participants chose to inform their partners face to face to ensure clarity or to respond immediately to questions or reactions:

‘I told him that the doctor said I have that disease again. I told him face-to-face.’ (Participant 4, 35-year-old female)

Furthermore, some participants opted to notify their partners telephonically, especially if a face-to-face conversation was impractical, some because of long-distance relationships and their responses were as follows:

‘I texted her and told her that they said I have an STI at the clinic.’ (Participant 17, 22-year-old male)

‘She is staying in rural areas, and I am staying here in Gauteng most of the time. I told her over the phone.’ (Participant 15, 39-year-old male)

## Discussion

The study explored PN practices and experiences of STI-diagnosed patients with their sexual partners in the Ekurhuleni East sub-district, Gauteng, South Africa. The results demonstrate that participants have varying understandings of STI transmission, prevention and symptom recognition, with some demonstrating appropriate knowledge and others holding considerable misconceptions. Participants identified condom use as the primary method of STI prevention, with recognition of symptoms such as burning during urination, itching, unusual discharge and genital warts. However, few participants demonstrated awareness of asymptomatic infections. This observation aligns with findings by Ricks et al.,^21^ which indicate that young adults generally have a strong awareness of condoms as a preventive measure against STIs. In addition, Akers et al.^22^ report that although teenagers recognise the importance of condom use, they often fail to consistently apply this knowledge in practice. Studies further highlight that while common symptoms such as burning during urination and discharge are widely recognised, awareness of asymptomatic STIs remains limited.^22,23^

The participants’ varied awareness of STI transmission aligns with findings from studies^24^ that point to widespread misconceptions within specific populations. For example, research has shown that many people, particularly in low-resource settings or where formal sexual health education is limited, hold inaccurate beliefs about STI transmission, associating it with personal hygiene or factors unrelated to sexual contact.^25^ Nonetheless, some participants did convey an awareness that unprotected intercourse is the leading way that STIs are spread, which is consistent with findings from previous international research.^26^ This shows that although public health messages, such as encouraging the use of condoms, are reaching some people, they might not be enough to eradicate common misconceptions.

Participants’ responses highlighted a spectrum of experiences with PN; some participants found the process challenging, while others encountered supportive or cooperative reactions. Overall, the narratives reveal a complex array of social and relational dynamics that influence the willingness to inform partners and partners’ responses to notification. McMahan and Olmstead^27^ similarly indicate that individuals with STIs experience a range of negative emotions related to disclosing their status. Reasons for disclosure included a sense of moral obligation, love for their partner and a desire for support, while reasons for withholding disclosure involved fears of negative partner reactions, potential rejection or breakup and beliefs about the lack of commitment.

Many participants reported difficulties and fear in notifying partners about STIs because of concerns about being blamed or accused of infidelity. Participants, particularly women, highlighted gender dynamics where they often anticipated judgement or being held responsible for STI exposure. These findings align with existing literature showing that fear of partner blame and accusations of infidelity are substantial barriers to PN, especially for women.^28^

While some participants reported positive reactions from their partners upon disclosure, many encountered resistances, particularly from partners unwilling to seek treatment or accompany them to the clinic. This reluctance delays essential care and adds strain to their relationships. Women, especially, anticipate negative reactions from their partners, such as criticism or accusations of infidelity, when disclosing their STI status.^28^ Societal expectations and stigma further heighten these concerns, contributing to a reluctance to initiate discussions about STIs.^29^ In line with the current study’s findings, research highlights the critical role that social dynamics and stigma play in shaping PN behaviours and their outcomes.

Various relationship dynamics were revealed by participants, such as IPV, infidelity and casual relationships, as influencers for the processes and experience of notifying partners about potential STI exposure. The participants’ responses revealed how trust issues, power imbalances and casual sexual practices often complicate disclosure and affect individuals’ approaches to health-seeking behaviours. Research has reported that power dynamics frequently influence disclosure, particularly in IPV circumstances. Victims may dread the negative consequences of exposing abuse, leading to silence and reluctance to seek treatment.^30^ Dougherty et al.^31^ indicated that women who had experienced recent sexual IPV had over two and a half times the odds of having foregone medical care compared with women with no experience. Research on social trust and health-seeking behaviours conducted in the Philippines revealed that social trust, particularly within family and community, enhances health-seeking behaviours, especially for stigmatised diseases.^32^ Furthermore, casual sexual practices can hinder health-seeking behaviours because people may not identify the need for medical help or are afraid of the stigma connected with their sexual history.^30^

Some participants reported experiencing or fearing violence from their partners following STI disclosure, while others noted instances of verbal abuse, blame and threats. These experiences align with Whitton et al.^30^ who found that the anticipation of negative reactions and stigma often prevents individuals from disclosing IPV experiences. Apiribu et al.^33^ similarly highlighted how the fear of judgement and adverse responses can be a significant barrier to disclosure. This compounded fear of violence and social stigma underscores the complexities and risks individuals face when considering whether to disclose sensitive health information. These instances of IPV highlight the heightened vulnerability faced by some individuals, particularly women. The findings are consistent with those of Decker et al.,^34^ that IPV can create a fear of PN, particularly among women. This fear can hinder effective PN, as IPV victims may avoid informing partners altogether.

In addition, Rosenfeld et al.^35^ find that many healthcare providers do not adequately consider IPV when recommending PN or expedited partner therapy, which can compromise patient safety. The study further highlighted a demand for enhanced provider training to assess IPV and its implications for STI management.^35^ In contrast, findings by Mathews et al.^36^ indicated that IPV was not identified as a barrier to PN.

infidelity-related trust concerns influenced participants’ responses to PN. Participants revealed how their partner’s infidelity led them to suspect their partners as the source of their STI. Nonetheless, some participants were accused by their partners of being the source of the infection. This is in line with Thompson and O’Sullivan,^37^ who found that individuals may assign blame for infidelity and STIs based on perceived control and intentionality, leading to accusations between partners. In addition, Pieluzek^38^ indicated that the emotional trauma from infidelity can lead to long-term psychological effects, including shame and stigma, impacting future relationships. These narratives demonstrate how trust loss and infidelity can introduce new levels of conflict and blame, affecting disclosure and treatment. This accords with Grøntvedt et al.^39^ who stated that infidelity is perceived as a significant threat, leading to an increased likelihood of breakup because of blame and a lack of forgiveness.

Participants also reported challenges related to disclosing STIs to casual or one-night-stand partners. They indicated that because of the temporary nature of these encounters, they prioritised treating themselves and did not inform the other party. Similarly, other participants noted that their relationships were casual and did not warrant notification, demonstrating a pattern of avoidance in casual relationships. This is in line with Nearchou et al.^40^ who found that participants often feel that casual relationships do not warrant disclosure, leading to prioritisation of personal treatment. In addition, Mathews et al.^36^ alluded that individuals in casual relationships are less likely to notify partners about STI exposure. The odds of PN decrease significantly in these contexts, as evidenced by a study.

### Limitations

The study focuses on a single sub-district and fewer healthcare facilities, which limits the generalisability of the findings to other areas, especially given the cultural and socio-economic diversity across the district, which includes a mix of urban and peri-urban communities, differences in education, income levels and cultural beliefs and practices related to sexual health. In addition, most of the participants were females, which may limit the generalisability of the findings to males or other gender groups, as their experiences with partner notifications may differ.

### Recommendations

The study explored PN practices and experiences of STI-diagnosed patients with their sexual partners. It is evident that there is a lack of awareness about STIs that further influences PN. Recommendations emerging from this study indicate that health programmes should focus on increasing awareness about STI transmission, prevention and symptoms. Community outreach and educational campaigns should target both young adults and key influencers, such as family members or peers, to ensure comprehensive understanding and reduce the stigma associated with STIs.

Interventions should be developed to support individuals in effectively notifying their partners about STI diagnoses. This could include providing counselling on managing emotional responses such as shame, anger and fear, as well as guidance on how to communicate health information to partners. In addition, addressing issues related to IPV and infidelity within STI prevention programmes is crucial.

## Conclusion

The broad scope of this study was to explore PN practices and experiences of STI-diagnosed patients with their sexual partners in the Ekurhuleni East sub-district, Gauteng province, South Africa. Therefore, this study highlighted the complex challenges and experiences of STI-diagnosed individuals with PN. Participants demonstrated different levels of knowledge about STI transmission, prevention and symptoms, with limited awareness of asymptomatic infections and persistent misconceptions. Social and relational dynamics, including fear of blame, infidelity, stigma and IPV, significantly influenced PN practices and willingness to disclose. While some participants encountered supportive reactions, many faced resistance and negative partner responses.
